# Role of Laboratory in Emerging Infectious Disease Control in Iran, Pasteur Institute of Iran, and national laboratory network

**DOI:** 10.1111/irv.13232

**Published:** 2023-12-10

**Authors:** Ali Maleki, Ehsan Mostafavi, Mehdi Fazlalipour, Mostafa Salehi‐Vaziri

**Affiliations:** ^1^ COVID‐19 National Reference Laboratory Pasteur Institute of Iran Tehran Iran; ^2^ Department of Influenza and other Respiratory Viruses Pasteur Institute of Iran Tehran Iran; ^3^ Rapid Response Team for Infectious Diseases Pasteur Institute of Iran Tehran Iran; ^4^ Department of Epidemiology and Biostatics, Research Centre for Emerging and Reemerging Infectious Diseases Pasteur Institute of Iran Tehran Iran; ^5^ WHO Collaborating Center for Reference and Research on Rabies Pasteur Institute of Iran Tehran Iran; ^6^ Department of Arboviruses and Viral Hemorrhagic Fevers (National Reference Laboratory) Pasteur Institute of Iran Tehran Iran

**Keywords:** COVID‐19, emerging infectious disease, Iran, laboratory network, SARS‐CoV‐2

## Abstract

Strengthening surveillance systems is a key aspect of outbreak response and was particularly important during the COVID‐19 pandemic. Respiratory pathogens spread rapidly, and laboratory capacity is key to monitoring the spread. Prior to the pandemic, Iran had established a rapid response team and laboratory network to provide identification, monitoring, and detection of emerging infectious diseases, but did not have the laboratory capacity to respond to COVID‐19. Following the announcement of the COVID‐19 pandemic, the rapid response team diverted all attention to supporting COVID‐19 surveillance. Iran built on the existing national laboratory infrastructure to incorporate SARS‐CoV‐2 surveillance into the response network. Based on existing international protocols, in‐house molecular diagnosis capacity was operationalized, and commercial controls and assays were acquired and validated to national standards. The first COVID‐19 laboratory was operational by January 25, less than 4 weeks before the initial detection of SARS‐CoV‐2 was announced. Assays and support were expanded and rolled out to form the COVID‐19 National Laboratory Network, which consists of 560 multi‐sectoral laboratories covering all provinces of Iran. The national laboratory network supports a wide range of operational capacities, including assay validation and protocol development, quality assurance, respiratory pathogen diagnosis and surveillance, and variant identification and assessment using multiple sequencing platforms. This network has supported the testing of over 55 million samples over the past 36 months using RT‐qPCR and has sequenced approximately 2200 samples across the country, contributing the data to international databases, including GISAID.

## 1 INTRODUCTION

Emerging infectious diseases can be defined as those that have recently appeared in a community for the first time or diseases whose prevalence or geographic range are increasing rapidly or will increase shortly.^1^ The incidence of emerging infectious diseases has increased globally in recent years and poses many challenges to the global community, as well as World Health Organization (WHO).^2^

Strengthening surveillance systems for accurate and timely identification of emerging diseases are key to guiding preventive and control measures.^3^ One of the main pillars of any surveillance system is the laboratory that plays an essential role in providing scientific data not only during an outbreak (for outbreak response and management) but before (for early detection of outbreaks and identification of etiological agents) and in between outbreaks (for monitoring the disease trend and evaluation of public health interventions). Coordinating the surveillance and laboratory systems in a country or a region can significantly improve the agility, accuracy, and comprehensiveness of laboratories. The unprecedented COVID‐19 pandemic showed that in the face of widespread epidemics and pandemics, networking should be considered one of the most important priorities.^4^ In Iran, Pasteur Institute of Iran (IPI) in collaboration with the Ministry of Health and Medical Education (MoHME) established the COVID‐19 National Laboratory Network in which 560 laboratories operate under the supervision of the COVID‐19 National Reference Laboratory (CNRL) at IPI. Here, we present the establishment process and the activities/tasks of this network.

## 2 ESTABLISHMENT OF THE COVID‐19 NATIONAL LABORATORY NETWORK IN IRAN

The foundation of the first COVID‐19 laboratory in Iran was laid several years before the emergence of COVID‐19, with the formation of the rapid response team for infectious diseases at the IPI. This action was taken in response to the need to create an agile structure for identifying, monitoring and detecting emerging infectious diseases (Table 1).^5^

**TABLE 1:** The main activity of Rapid Response Team of Institute Pasture of Iran.

The Task Description of Rapid Response Team, IPI	
1	Monitoring potential threats in the field of infectious diseases, especially emerging and re‐emerging pathogens
2	Designing and providing molecular laboratory diagnostic tests for diseases with the potential to cause a pandemic
3	Improving the knowledge of team members by participating in national and international training courses, including the outbreak investigation courses of the World Health Organization
4	Using the capacity of national reference laboratories located in the IPI to strengthen their capabilities
5	Applying the knowledge and skills of experts from various fields including virology, bacteriology, parasitology, medicine, epidemiology, entomology and biotechnology
6	Establishing communication and gaining support from stakeholders in the Ministry of Health, including the Iranian CDC and Health Reference Laboratory

From its establishment until the COVID‐19 pandemic, the team has taken effective measures to deal with outbreaks of various infections, such as *Crimean‐Congo Hemorrhagic Fever viru*s (CCHFV), *Middle East Respiratory Syndrome Coronavirus* (MERS‐CoV), and rodent‐borne infections like *Francisella tularensis* or *Yersinia pestis*.

**FIGURE 1:**
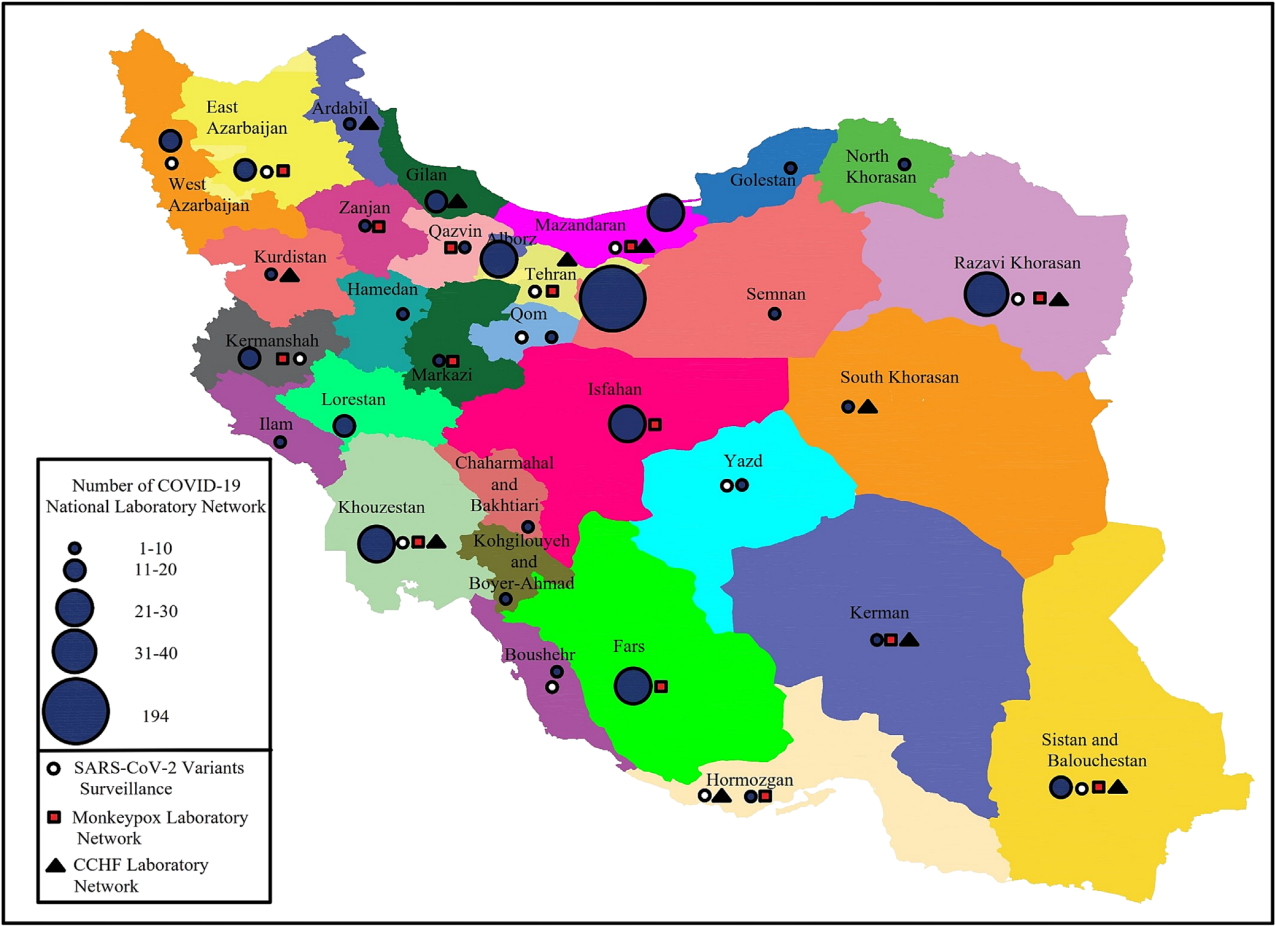
Distribution of COVID‐19, CCHF and MPox laboratories in the laboratory network in Iran. Colored circles indicate the COVID‐19 laboratories; the hollow circles represent centers with sequencing capacity to identify SARS‐CoV‐2 variants; the squares represent the MPox laboratory and the triangles represent the CCHF laboratory.

Shortly after the news of the detection of a novel coronavirus in China on 31 December 2019, the rapid response team decided on January 18, 2020, to use all available capacities to launch diagnostic tests for the emerging virus (named 2019 novel coronavirus [2019‐nCoV] at that time) as soon as possible. In this regard, the following several actions were taken: (1) optimizing the pan‐coronavirus RT‐PCR test that had been designed in 2015^6^; (2) designing diagnostic tests based on the published protocols of Corman et al^7^ and Hong Kong University^8^; (3) sending a request for clinical kits and positive control to WHO, Pasteur Institute networks, and EVAg; and (4) ordering available commercial kits in the market including PrimerDesign Ltd., UK (COVID‐19 genesig® Real‐Time PCR assay, detecting RdRp gene).^5^ Subsequently, the first COVID‐19 laboratory started working as the national reference laboratory on January 25, 2020, at the IPI, and the readiness to receive suspected samples was formally announced to the Ministry of Health. After the detection of the first case of COVID‐19 in Iran on February 18, 2020, the establishment of a laboratory network was assigned to the IPI by the Ministry of Health. Within a short time, the COVID‐19 National Laboratory Network was established and expanded so that currently 560 laboratories are under the supervision of CNRL and the health reference laboratory in all the provinces of the country (Figure 1). During the 3 years of the COVID‐19 pandemic, the COVID‐19 national reference laboratory has implemented key activities in the network, which include the following.

### 2.1 Validation of diagnostic assays

In the first months of the COVID‐19 pandemic in Iran, the CNRL was the only reference for protocol development and evaluation of nucleic acid extraction, real‐time PCR, and rapid antigen tests, and over time, this task was assigned to the Food and Drug Control Reference Laboratories.^5^ Due to the unprecedented situation of COVID‐19 pandemic, we employed the emergency use authorization (EUA) strategy for diagnostic kit evaluation. Briefly, regarding real‐time PCR kits, panels of SARS‐CoV‐2‐positive clinical specimens with different viral loads (Ct values) and also negative samples that were tested by the WHO‐approved kits were used. Kits with the ability to correctly identify 95% of panel samples were approved. The RNA extraction kits were evaluated with similar strategy. For rapid antigen tests, according to the WHO recommendation,^9^ any kit with ≥80% sensitivity and ≥97% specificity compared to real‐time PCR assay was approved.

### 2.2 Roll‐out of diagnostic kits to laboratories in the network

At the beginning of the COVID‐19 pandemic, all commercial diagnostic kits, whether domestic or foreign production, were sent to the IPI, and these kits were distributed in the network after quality assessment and approval from the CNRL. Currently, the distribution of diagnostic kits in the network is performed by the health reference laboratory and Heyat Omanaye Arzi (the office responsible for international procurements of the MoHME).

### 2.3 Organization of external quality assessments (EQAs)

To guarantee the quality of laboratory tests throughout the network, a strict external quality assessment (EQA) for the detection of SARS‐CoV‐2 infection by real‐time PCR assay was designed, and all laboratories were required to participate in the assessment every 6 months. In this program, five to six unknown samples, including inactivated clinical SARS‐CoV‐2‐positive samples with different viral loads, clinical SARS‐CoV‐2 negative samples and cell‐free viral transport medium (VTM) are sent to each laboratory. Every laboratory is required to perform the test within 30 h of receiving the samples.

If the laboratory fails in the EQA, its license is suspended, and the laboratory must participate in the EQA again after implementing corrective actions. In addition to EQA, periodic audits and course inspections are conducted by the CNRL's experts. The CNRL itself participates in WHO EQAs.

### 2.4 Development of technical guidance documents

Since the beginning of the pandemic, the CNRL has played a key role in the development and revision of all protocols related to laboratory diagnosis and monitoring of the virus circulation including the national standard protocol for detection of acute SARS‐CoV‐2 infection by real‐time PCR and antigen detection assays, application of serological assays, SARS‐CoV‐2 variant surveillance, and biosafety requirements.

### 2.5 Implementation of respiratory viral infections surveillance

Coinfection of SARS‐CoV‐2 and influenza viruses was considered a serious challenge in late 2020. Therefore, CNRL designed multiplex real‐time PCR for simultaneous detection of COVID‐19 and influenza A and B and distributed in the network before the beginning of the influenza wave in 2020. Our multiplex assay could target two N gene regions of SARS‐CoV‐2, M gene of the influenza A virus, NS1 gene of the influenza B virus, and the human RNase P gene as an internal control. Fortunately, because of lockdowns, infection control measures, and social distancing, the spread of influenza and other respiratory viruses was reduced during the first year of the COVID‐19 pandemic.^10^ However, it resulted in lower levels of immunity against these viruses due to interruption in vaccination programs and decreased natural immunity, which in turn led to epidemics of respiratory viruses following the termination of social distancing in 2021.^11^, ^12^ In response to prevent future outbreaks of other respiratory viruses other than SARS‐CoV‐2, the national reference laboratory employed multiplex assays for the detection of SARS‐CoV‐2, influenza A and B viruses, respiratory syncytial virus (RSV), HCoV‐OC43, HCoV‐HKU1, HCoV‐229E, HCoV‐NL63, metapneumovirus, adenovirus, parainfluenza 1‐2–3 viruses, and human bocavirus 1–2–3 viruses. In addition to providing comprehensive data about the circulating respiratory viruses to the Iranian CDC for adopting better control measures, employing multiplex assays could help hospitals to better triage and treat the patients.^13^

### 2.6 SARS‐CoV‐2 variants surveillance

Iran was one of the pioneer countries that launched the genomic surveillance system for SARS‐CoV‐2. IPI started monitoring the SARS‐CoV‐2 mutation from March 2020, by partially sequencing the S gene using a Sanger assay. CNRL developed some in‐house qRT‐PCR tests as variants of concern (VOCs) screening kits to examine signature mutations in VOCs like 69/70del, 144del, and L452R. As a confirmative assay, the sequencing assay must validate the qRT‐PCR VOC screening assay results. Sanger sequencing was used to perform partial sequencing on a percentage of positive tests for the VOC screening qRT‐PCR. Primers for different regions of the spike and ORF1 genes were designed and provided. Based on the type of VOC, a specific region of the SARS‐CoV‐2 genome, particularly the receptor binding domain (RBD), was amplified and sequenced to confirm the results of VOC screening qRT‐PCR kits. Thus, CNRL not only confirmed the primary results for reporting but also figured out which subvariants were in circulation. The result of partial sequencing is reliable at the time, but due to the evolving mutation algorithm in the particular genomic region sequenced, it might be challenging to analyze in the future. For developing countries without an NGS system for the surveillance of newly emerging or reemerging infectious diseases, this procedure is very functional and well suited.

IPI developed the surveillance system based on two main branches, routine and unusual events surveillance. These two strands of evidence should be brought together in a timely fashion to provide a broad understanding of viral evolution and its potential impact on disease control to guide public health response. The routine surveillance has been performed weekly by randomly selecting a proportion of samples sent to CNRL. The unusual events surveillance was designed to monitor genetic variations in unusual cases and conditions including vaccine breakthrough, reinfection, immunocompromised patient, zoonotic transmission, poor response to therapeutics, unusual clinical presentations, travel history to a region with a high incidence of VOCs or variants of interest (VOIs), and diagnostics test discrepancies.

IPI received its first NGS machines, GridION and MinION, in late 2021 from Oxford Nanopore Technologies (ONT, UK). These two NGS machines were donated by the WHO, along with the series of Midnight kits (ONT, UK) for whole genome sequencing of SARS‐CoV‐2. During 1 month, additional required equipment and materials had been provided, and the optimization process started. CNRL had been receiving respiratory samples, and oropharyngeal swabs in VTM, from the different diagnosis laboratories and hospitals, so it was able to sequence the SARS‐CoV‐2 from respiratory samples, but it was not enough. If IPI wanted to monitor the variations in SARS‐CoV‐2 and their prevalence, it had to have samples from widespread locations. Therefore, IPI decided to design a laboratory network for receiving the respiratory network throughout the country to cover more geographical regions (Figure 1). To make the process easier, IPI selected 10 laboratories from the national laboratory network developed at the beginning of the COVID‐19 pandemic in Iran. These laboratories were distributed throughout the country with different geocultural circumstances which ensure that the samples represent different Iranian communities. The recommendation was for the selected laboratories to send 30 samples per week. The qualified samples with a cycle of threshold (Ct) less than 25 were chosen for sequencing. After data analysis and reporting, the sequences were submitted to Global Initiative on Sharing Avian Influenza Data (GISAID).

Recently, with the emergence and circulation of some new immune‐escaping subvariants of Omicron, such as BQ.1.1, BF.7, XBB.1.5, and CH.1.1 in the world, the role of SARS‐CoV‐2 variants surveillance in Iran became important one more time. Despite the predominance of some of the abovementioned Omicron subvariants in the United States and some European countries during the current wave of COVID‐19, Iran did not experience any wave at that time. SARS‐CoV‐2 variant surveillance showed less than 5% prevalence for each XBB.1.5, BQ.1.1, and CH.1.1 or less than 10% of the total of them (unpublished data).

In addition, the CNRL developed proxy assays to monitor VOCs. The standard method of variants identification is based on the sequencing of the viral genome, but advanced sequencing equipment was not available in the majority of laboratories in the network. So, the CNRL designed real‐time PCR‐based kits for the detection of variant‐specific mutations to accelerate the monitoring of VOCs in the country.

So far, approximately 2200 SARS‐CoV‐2‐positive samples have been subjected to sequence analysis, and the results submitted to GISAID. These submission have been included the partial sequencing by Sanger assay and mostly, the whole genome sequencing by NGS system.

### 2.7 Biobanking of clinical samples

As a result of the monitoring of COVID‐19 variants and respiratory viruses, positive clinical samples of Alpha, Beta, Delta, and Omicron (and their sublineages, including BA.1, BA.2, BA.4, BA.5, BQ.1, and XBB.1) SARS‐CoV‐2, and several respiratory viruses were identified. These viruses were subjected to sequencing and clinical samples as well as the genetic materials stored in the CNRL.

## 3 EXPANDING THE CAPACITY OF THE NATIONAL LABORATORY NETWORK (FROM COVID‐19 LABORATORY NETWORK TO THE MOLECULAR HEALTH LABORATORY NETWORK)

Despite the enormous challenges posed by the COVID‐19 crisis in Iran, which has led to the death of more than 144,000 people, the establishment of the national COVID‐19 laboratory network has been a great achievement. This infrastructural capacity can be used for the molecular diagnosis of other emerging infectious diseases besides COVID‐19. Preliminary measures have been taken to expand the COVID‐19 laboratory network into a molecular health laboratory network. For example, in response to the MPox (formerly known as monkeypox) outbreak in May 2022, the rapid response team for the infectious disease of IPI designed a molecular diagnostic kit for the screening of orthopoxviruses and distributed it in 14 selected laboratories throughout the network (Figure 1). The first imported case of MPox in Iran was detected by the screening kit in one of the network's laboratories in southwest Iran and then confirmed by IPI using confirmatory MPox real‐time PCR and sequencing (unpublished data).

Additionally, kit procurement and the implementation of training courses for the establishment of Crimean‐Congo Hemorrhagic Fever (CCHF) molecular diagnosis have been carried out in 10 selected laboratories of the network by the national reference laboratory of arboviruses and Viral hemorrhagic fevers of IPI (Figure 1). In response to the recent identification of *Aedes aegypti*, the main vector of dengue, chikungunya, and Zika viruses in Hormozgan province, southern Iran,^14^ kit procurement and the implementation of training courses for molecular diagnosis of dengue, chikungunya, and Zika viruses have been done in Hormozgan University of Medical Sciences' laboratory.

The network is ready to provide health services to the country and is willing to participate in international collaborations, especially in the EMRO region. In this regard, IPI has already cooperated with WHO‐EMRO in several programs, including technical support of Afghanistan and Iraq for the CCHF surveillance system in 2017 and 2018, respectively.

## 4 CONCLUSION

Considering the increasing number of emerging and re‐emerging diseases, it is necessary to strengthen the performance potential of reference laboratories. This preparation must be in the field of trained staff, supplying equipped devices, and developing diagnostic and control capabilities at the country level. By increasing the number of reference centers and laboratories and coordinating CDC headquarters and management, this capacity can be helpful in controlling diseases that spread rapidly in society and lead to damaging pandemics in crises such as COVID‐19 pandemic.

## AUTHOR CONTRIBUTIONS

**Ali Maleki:** Substantial contributions to conception, design of the study, and drafting the manuscript. **Ehsan Mostafavi:** Drafting the manuscript and revising it critically. **Mehdi Fazlalipour:** Drafting the manuscript and revising it critically. **Mostafa Salehi‐Vaziri:** Substantial contributions to conception, design of the study, and drafting the manuscript.

## CONFLICT OF INTEREST STATEMENT

None to declare.

## ETHICS STATEMENT

None to declare.

## Data Availability

The data that support the findings of this study are available from the corresponding author upon reasonable request.
